# On the Present State of Medicine in Prussia

**Published:** 1836-01

**Authors:** J. F. C. Hecker

**Affiliations:** Professor of Medicine in the University of Berlin


PART FOURTH.

### Medical Intelligence.

### REPORTS OF FOREIGN MEDICINE AND MEDICAL INSTITUTIONS.

One of the principal objects contemplated by the Editors in establishing this Journal, was to make the members of the profession in Great Britain more fully acquainted with the state of medical science in other countries. For this purpose, in addition to the more common and obvious sources of information open to them in the productions of the press, of which they determined fully to avail themselves, they conceived that it would be at once extremely interesting and useful, if they could obtain a series of Original Reports of the present State of Medicine, and of the Medical Institutions, in Foreign Countries, concisely drawn up by gentlemen belonging to and resident in them, and consequently well acquainted with their actual condition: and they are happy to say, that their endeavours to obtain this kind of information have met with the most cordial reception from their brethren abroad. The task has been cheerfully undertaken by men of great talent and acquirement, and of the first rank in the profession; and the Editors hope to present to their readers the result of their labours, in an unbroken series of communications, in the consecutive numbers of this Journal. To the first of these Reports, given below, and for which they are indebted to one of the most accomplished physicians in Europe, the Editors hope they may be permitted to refer, as sufficient evidence of the great importance of a collection of similar documents relating to all civilized countries.

### ON THE PRESENT STATE OF MEDICINE IN PRUSSIA;

By J. F. C. Hecker, M.D., Professor of Medicine in the University of Berlin.

### FIRST REPORT.

### *Of the Medical Institutions and the Medical Profession.*

#### THE UNIVERSITIES.

Prussia, with a population of 13,510,030, possesses six Universities, each having four Faculties; viz. those of Berlin, Bonn, Breslau, Greifswald, Halle, and Königsberg. The Medical Faculties in these Universities have all the same general arrangements, in accordance with the common system of instruction established in Prussia, but differ in regard to the number of professors and the facilities which they afford for study. In conformity with their statutes, they not only supply the means of instruction in medicine, but afford opportunities for the cultivation and advancement of the whole of the collateral sciences.

#### I. THE UNIVERSITY OF BERLIN.

The University of Berlin is the most considerable of the whole. It was founded in the year 1809, at a period when Prussia, reduced to half her dominions, was preparing her regeneration, through a more powerful development of her moral strength.

From its very foundation, this university was numerously attended; and, in the year 1834, reckoned on its books 1800 students of all classes, viz. 1294 natives, and 506 foreigners. Of these, 303 studied medicine; of whom, 237 were natives, and 66 foreigners. This was a diminution, by no less than 104, of the number of medical students who attended the university in the preceding year, 1833; there being then on the books 407. This decrease does not depend on any local causes connected with the university, but is explained by the fact that, in Germany generally, the number of students has been regularly decreasing during the last five years. This diminution is more conspicuous in the theological and juridical faculties than in the medical; and, in this last, it is less in the Prussian universities than elsewhere, being indeed very inconsiderable, when the whole number of medical students is considered. The exclusive cause of this falling off in the number of students, more striking perhaps in other states, is the sudden increase of the population in most of the German states, and the disproportion consequently produced between the present and the immediately preceding generation; which circumstance is very adverse to the advancement of young men in general, and forces many to betake themselves to occupations in trade and handicraft. The increase of the population in Prussia alone, since the year 1815, has been two and a half millions.

In the medical faculty of Berlin there are sixteen ordinary professors, (professores publici ordinarii;) ten extraordinary (professores publici extraordinarii;) and fifteen private teachers, or lecturers, (doctores privatim docentes;) so that, taken altogether, the medical instructors in the university amount to forty-one.

The following are the names of the professors, and the subjects belonging to their respective chairs:

*Ordinary Professors.*

| | |
|---|---|
| 1. C. W. Hufeland, . | Clinical Medicine.* |
| 2. Link, . . . . | Botany, Natural History, and Pharmacology. |
| 3. Von Gräfe, . . . | Surgery. |
| 4. Rust, . . . . | Surgery. |
| 5. Horkel, . . . . | Physiology. |
| 6. Horn, . . . . | Pathology and Therapeutics, and Mental Diseases. |
| 7. Bartels, . . . | Special Pathology and Therapeutics. |
| 8. Busch, . . . | Midwifery. |
| 9. Fr. Hufeland, . . | Semeiotics, general and special Pathology and Therapeutics. |
| 10. Osann, . . . . | Materia Medica. |
| 11. Wagner, . . . | State Medicine. |
| 12. Müller, . . . . | Anatomy and Physiology. |
| 13. Schlemm, . . . | Anatomy. |
| 14. Schultz, . . . . | Physiology and Botany. |
| 15. Hecker, . . . . | History of Medicine, general and special Pathology and Therapeutics. |
| 16. Jüngken, . . . | Diseases of the Eye. |

It is here to be observed, that the above statement of the offices of the professors (as is likewise the case in the other universities,) is only to be taken generally; any one of them may give lectures in any department which he chooses. A more fixed arrangement relative to the Nominal Professorships (so called) is expected to be made very shortly.

*Extraordinary Professors.*

| | |
|---|---|
| 1. Reich, . . . . | Practical Medicine. |
| 1. Kluge, . . . . | Surgery. |
| 3. Casper, . . . | State Medicine. |

* Hufeland no longer delivers public lectures, but occasionally gives clinical lectures in the Polyclinic of the university.

4. Ehrenberg, . . Physiology and the Natural Sciences.
5. Kranichfeld, . . Diseases of the Eye.
6. Eck, . . . . Physiology.
7. Wolff, . . . . Clinical Medicine.
8. Dieffenbach, . . Surgery.
9. Trüstedt, . . . Clinical Medicine and Surgery.
10. Froriep, . . . Pathological Anatomy.

*Private Teachers.*

| | | | |
|---|---|---|---|
| 1. Reckleben, | 5. Gräfe, | 9. Ascherson, | 13. Isensee, |
| 2. Barez, | 6. Ideler, | 10. Nicolai, | 14. Troschel, |
| 3. Oppert, | 7. Angelstein, | 11. Phöbus, | 15. Mitscherlich. |
| 4. Romberg, | 8. Dann, | 12. Wilde, | |

All the ordinary professors, and four of the extraordinary professors, receive salaries; the joint amount of which is 15,450 rix dollars, (about 2,300*l.* English.)

*Medical and Scientific Establishments.*

These are more numerous and varied than in any of the other Prussian universities. The following are immediately connected with the university:

1. A Medical Clinic, under the direction of Bartels; the annual expense of which is 1500 dollars, (225*l.*)
2. A Surgical Clinic, under Rust.
3. An Ophthalmological Clinic, under Jüngken.
4. A Midwifery Clinic, under Kluge.
5. A Clinic for Syphilitic Diseases, under the same professor.
6. A Clinic for Children's Diseases, under Barez.
7. A Clinic for Mental Disorders, under Ideler.

All these clinical institutions are held in the Charité Hospital, (Charité-Krankenhaus,) and are maintained by its revenues. In addition to the foregoing, the university has also the following institutions attached to it:

8. A Clinic for Surgery and Diseases of the Eye, under Von Gräfe, (Ziegelstrasse, No. 6,) which costs annually 6,700 dollars, (1000*l.*)
9. A Polyclinic for Ambulatory Practice,* under Osann, in the university buildings, with an expense of 2000 dollars, (300*l.*)
10. Another Polyclinic, under Trüstedt, (Ziegelstrasse, No. 6.) This is dependent on the administration of the Charité, and admits, for pay, a small number of patients confined to their beds, after the manner of the French *Maisons de Santé.*
11. The Obstetric Clinical Institution, under Busch. This occupies a handsome separate building (Dorotheentrasse, No. 1,) and has 5,468 dollars (820*l.*) for its annual support.
12. A Medical Clinic for Surgeons, in the Charité Hospital, under Wolff.
13. An institution for practical instruction in State Medicine, (Medical Jurisprudence and Medical Police,) has existed for some years, under the direction of Wagner. This establishment takes notice of such cases and instances in the department of medical jurisprudence as occur in Berlin, including cadaveric inspections, &c.

The following establishments, valuable in themselves, and much frequented by the students, are well deserving notice; and, with the enumeration of these, this brief account of the Berlin University shall close.

14. The Anatomical Theatre and Museum, under the superintendence of Müller (director,) and Schlemm (prosector). For the support of both establishments, 3,167 dollars (475*l.*) are set apart.
15. A very valuable collection of surgical and obstetrical instruments and bandages, kept up by an annual expenditure of 430 dollars, (64*l.*) under the management of Kluge.

* For visiting patients at their houses, like our Dispensaries.

16. The Botanic Garden, in New Schöneberg, with a yearly expenditure of 11,228 dollars, (1,684*l.*;) and the University Botanic Garden, with 500 dollars, (75*l.*:) both under Link.

17. The Great Herbarium, in New Schöneberg, with 1200 dollars, (180*l.*)

18. The Mineralogical Cabinet, with 1,520 dollars, (228*l.*) under Weiss.

19. The Zoological Museum, with 2,294 dollars, (344*l.*,) under Lichtenstein.

20. The Collection of Mathematical and Philosophical Apparatus; 500 dollars, (75*l.*)

21. The Royal Library, with 15,102 dollars, (2,265*l.*,) under Wilken.

22. The Institution for Veterinary Medicine. This is a very large and complete establishment, unconnected with the university, but much frequented by the students.

II. THE UNIVERSITY OF BONN.

This is the newest of all the Prussian universities, having been founded only in 1818. Since its establishment, it has enjoyed a constantly increasing prosperity. In the year 1834, there were at this university 816 students; of whom, 710 were natives, and 106 foreigners; of the whole number, 156 (138 natives, 18 foreigners,) were students of medicine.

The Medical Faculty comprehends eleven ordinary and one extraordinary professors, and two private lecturers. The following are the names of the present professors, and the departments in which they teach:

*Ordinary Professors.*

| | |
|---|---|
| 1. Harless, . . . | Theory of Medicine. |
| 2. Nasse, sen., . . | Pathology, Therapeutics, and Clinical Medicine. |
| 3. Stein, . . . . | Midwifery. [Has not delivered any lectures for a long time.] |
| 4. Windischmann, sen., | Theoretical Medicine. [He belongs also to the Faculty of Philosophy.] |
| 5. Mayer, . . . . | Anatomy and Physiology. |
| 6. Bischoff, . . . | Materia Medica. |
| 7. Eunemoser, . . | Theoretical and Psychical (Mental) Medicine. |
| 8. Naumann, . . . | Special Pathology and Therapeutics. |
| 9. Wutzer, . . . | Surgery. |
| 10. Kilian, . . . . | Midwifery. |
| 11. Weber, . . . . | Anatomy. |

*Extraordinary Professor.*

| | |
|---|---|
| 12. Albers, . . . . | Pathology and Therapeutics. |

*Private Lecturers.*

1. Nasse, jun. 2. Windischmann, jun.

The joint annual salaries of the professors amount to 11,300 dollars, (1,695*l.*)

The university has three clinical establishments:

1. A Medical Clinic, under Nasse, sen., with an annual income of 4,017 dollars, (602*l.*)

2. A Surgical and Ophthalmological Clinic, under Wutzer, with 4,091 dollars, (613*l.*)

3. An Obstetrical Clinic, under Kilian, with 1,773 dollars, (465*l.*)

In all the three establishments, the Polyclinic is adopted, to the great increase of their usefulness. Indeed, in all the Prussian universities due attention is paid to the Polyclinic, and with much advantage; it being very important that the student should be introduced into the private dwellings of the sick, and thereby be made acquainted betimes with the many obstacles which the exigencies of civil life, beyond the walls of hospitals, oppose to the practice of medicine.

4. The Anatomical Theatre and Museum, under the superintendence of Mayer (the director), and Weber (prosector), with an expenditure of 1500 dollars (225*l.*) per annum.

5. The Collection of Surgical Instruments and Bandages, in the care of Wutzer. This is connected with the Surgical Clinic, and is maintained from its funds.

Besides the foregoing more strictly medical institutions, this university is richly provided with establishments and collections for the study of the natural sciences connected with medicine. Of these may be mentioned,

1. The Zoological and Mineralogical Museum, under Goldfuss and Nöggerath; together having an annual expenditure of 1,150 dollars, (172*l.*)

2. The Botanic Garden, under Treviranus and Nees von Esenbeck, with 2,560 dollars, (384*l.*)

3. A Pharmacological Cabinet, under Bischoff, with 50 dollars, (7*l.* 10*s.*)

4. The University Library, under Welcker, with 4621 dollars, (693*l.*)

Besides the above, there are several other smaller establishments belonging to the university, which need not be mentioned.

There exists in Bonn an institution for the cultivation of the whole of the natural sciences, of which Nöggerath, Von München, Treviranus, Goldfuss, and G. Bischof, are the chief managers, and which has hitherto exhibited great activity and been of much use.

### III. THE UNIVERSITY OF BRESLAU.

This university was established in the year 1811, on the suppression of that of Frankfurt on the Oder. In 1834, it had 829 students; of which number, 812 were natives, and 11 foreigners: of these, 107 belonged to the faculty of medicine, viz. 101 natives and 6 foreigners.

*Ordinary Professors.*

| | |
|---|---|
| 1. Remer, . . . | Theory of Medicine and Clinical Medicine. |
| 2. Benedict, . . . | Surgery and Diseases of the Eye. |
| 3. Otto, . . . . | Anatomy and Physiology. |
| 4. Wendt, . . . | Practical Medicine. |
| 5. Purkinje, . . . | Physiology. |
| 6. Hentschel, . . . | Botany, History of Medicine, &c. |
| 7. Betschler, . . . | Midwifery. |

*Extraordinary Professors.*

| | |
|---|---|
| 1. Barkow, . . . | Anatomy. |
| 2. Goeppert, . . . | Botany and Natural History generally. |

The annual amount of the salaries of the professors is 6,650 dollars, (997*l.*)

*Private Lecturers.*

1. Hemprich; 2. Seidel; 3. Wentzke; 4. Remer, jun.; 5. Küstner.

*Medical Establishments.*

1. The Anatomical Theatre and Museum, under Otto (director), and Barkow (prosector), with an annual expenditure of 1898 dollars, (334*l.*)

2. The Museum of Natural History, under Gravenhorst, with 868 dollars, (130*l.*)

3. The Medical Clinic, under Remer, with 2,569 dollars, (385*l.*)

4. The Surgical Clinic, under Benedict, with 2000 dollars, (300*l.*)

5. The Obstetrical Clinic and Polyclinic, under Betschler, with 600 dollars, (90*l.*) Connected with this is a School for the instruction of midwives, with an income of 400 dollars, (60*l.*)

6. The Botanic Garden, under the direction of Nees von Esenbeck, sen., formerly of Bonn, perpetual president of the Leopoldine Academy. This garden has very extensive grounds, and is kept up at an annual expense of 2,610 dollars, (391*l.*)

7. The Mineralogical Cabinet, with 200 dollars (30*l.*), under Glocker.

8. The Collection of Philosophical Instruments, with 80 dollars (12*l.*), under Pohl.

9. The Chemical Laboratory, with 372 dollars (55*l.*), under Fischer.

10. A Physiological Institution, under Purkinje, with no fixed income.

11. The University Library, with 5,130 dollars (769*l.*), under Wachler.

### IV. THE UNIVERSITY OF GREIFSWALD.

This university, situated in Pomerania, is one of the oldest in Prussia, having been founded in the year 1456. Although possessed of large revenues from ancient endowments, Greifswald has never raised itself to any importance, and is at this time the most inconsiderable in the kingdom.

In 1834, the number of students was 187; of whom, 172 were natives, and 15 foreigners; and of these, 60 belonged to the faculty of medicine, viz. 54 natives, and 6 foreigners.

*Ordinary Professors.*

1. Schultze, . . . Anatomy and Physiology.
2. Bernt, . . . . Practical and Clinical Medicine.
3. Mandt, . . . . Surgery.*
4. Seifert, . . . . Pathology and Therapeutics.

Their united salaries amount to 4,163 dollars, (619*l.*)

*Private Lecturers.*

1. Laurer (prosector); 2. Kneip; 3. Biel.

This university possesses suitable institutions and means for the study of the medical sciences. The following are the principal:

1. A stationary and ambulatory Clinic, under Berndt, with an expenditure of 850 dollars, (127*l.*)

2. A Surgical Clinic, under Mandt.

3. An Obstetrical Clinic, connected with a School for the instruction of midwives, under Berndt, with 380 dollars, (57*l.*)

4. A Botanic Garden, under Hornschuch, with 1,010 dollars, (151*l.*)

5. An Anatomical Theatre and Museum, under Schultze, with 677 dollars, (101*l.*)

6. An University Library, under Schildener, with 1,715 dollars, (257*l.*)

7. A Zoological Museum, under Hornschuch, with 1000 dollars, (150*l.*)

8. A Chemical Institution, under Hünefeld, with 160 dollars, (24*l.*)

9. A Cabinet of Philosophical and Mathematical Instruments, under Tillberg, with 60 dollars, (9*l.*)

### V. THE UNIVERSITY OF HALLE.

This university was founded in the year 1694, and, throughout the whole of the eighteenth century, was one of the most distinguished for medical science. Beginning with Stahl and Frederick Hoffmann, this university subsequently possessed many eminent professors. It is now, however, less considerable than many others in Germany.

In 1834, the number of students were 752; of whom, 618 were natives, and 134 foreigners; and of these, 114 belonged to the faculty of medicine, viz. 79 natives, and 35 foreigners.

*Ordinary Professors.*

1. Krukenberg, . . Practical and Clinical Medicine.
2. Friedlander, . . Theoretical Medicine.
3. Niemeyer, . . . Midwifery.
4. D'Alton, . . . Anatomy.
5. Blasius, . . . Surgery.

*Extraordinary Professors.*

1. Schweigger-Seidel, Chemistry.
2. Hohl, . . . . Surgery.

Their salaries amount to 9,022 dollars, (1,350*l.*)

* This professor is now absent, and will probably take an appointment in Russia.

The following are the principal establishments, with their yearly endowments:

1. The University Library, under Voigtel, with 2,820 dollars, (423*l.*) This is very valuable.
2. The Botanic Garden, under Von Schlechtendal, with 1,090 dollars, (163*l.*) A valuable establishment.
3. The Obstetrical Institution, under Niemeyer, with 1000 dollars, (150*l.*)
4. The Medical Clinic, under Krukenberg, with 3,040 dollars, (456*l.*)
5. The Surgical Clinic, under Blasius, with 1,210 dollars, (181*l.*)
6. The Anatomical Theatre and Zootomical Museum, under D'Alton.
7. The Zoological Museum, under Nitzsch. This establishment is united with the Zootomical, and both have a revenue of 1,470 dollars, (220*l.*)
8. The Physico-chemical Cabinet and Laboratory, under Schweigger, with 520 dollars, (78*l.*)
9. The Mineralogical Cabinet, under Germar, with 280 dollars, (42*l.*)
10. The Pharmaceutical Institution, under Schweigger-Seidel.
11. A Polyclinic, of considerable extent.

The celebrated Anatomical Museum of Meckel, formerly an ornament of this university, is private property, not having been as yet purchased by the government.

### VI. THE UNIVERSITY OF KÖNIGSBERG.

This university was founded in 1544, in the time of the religious wars, and still retains its ancient privilege of admitting only to its professorships such as profess the Protestant faith. Owing to its remoteness, it is chiefly attended by the natives of the province (of Prussia), and only has a small list of students, In 1834, these amounted to 422, (384 natives, and 38 foreigners;) of whom, 83 (70 natives, and 13 foreigners,) belonged to the faculty of medicine.

*Ordinary Professors.*

1. Burdach, sen., . . . Physiology.
2. Sachs, . . . . . Practical Medicine.
3. Klose, (formerly of Breslau,) Clinical Medicine.
4. Rathke, . . . . . Anatomy and Physiology.
5. Seerig, . . . . . Surgery.

*Extraordinary Professors.*

1. Hayn, . . . . . Midwifery.
2. Dietz, . . . . . Practical Medicine.

These have salaries, amounting in all to 5,318 dollars, (797*l.*)

*Private Lecturers.*

1. Richter; 2. Cruse; 3. Burdach, (prosector:) all advantageously known in medical literature.

The following are the different establishments:

1. The University Library, under the philologist Lobesk, with a yearly revenue of 3,590 dollars, (538*l.*)
2. The Botanic Garden, under Meyer, with 2,100 dollars, (315*l.*)
3. The Anatomical Theatre, under Rathke, with 1,180 dollars, (172*l.*)
4. The Medical Clinic. The nominal director of this is at present Klose, in the room of Elsner, who died in 1834; the duties of the office being fulfilled by the assistant physician, Dietz. The annual expenditure is 2,300 dollars, (345*l.*)
5. The Lying-in Hospital, with which is connected an institution for the instruction of Midwives, under Hayn, with 220 dollars, (33*l.*)
6. The Surgical Clinic, under Seerig, with 2,455 dollars, (368*l.*)
7. The Medical Polyclinic, under Sachs, with 200 dollars, (30*l.*)
8. The Zoological Museum, with 1000 dollars (150*l.*), the directorship of which is at present vacant.
9. The Mineralogical Cabinet, with 100 dollars, (15*l.*)
10. The Cabinet of Philosophical Instruments, with 158 dollars, (23*l.*) Both these are under the direction of Neumann.

From the preceding statement it results, that, in the whole Prussian states, there are somewhat more than eight hundred medical students, and who are instrncted by ninety-three teachers,—viz. forty-seven ordinary professors, seventeen extraordinary professors, and twenty-nine private lecturers.—These students, with very few exceptions, all obtain the doctorate; and it is characteristic of the course of study in the Prussian universities, that the students are fitted for their particular branch by previous scientific instruction, and that they, for the most part, belong to the better classes of society. It is a law that no one can be admitted to examination for the degree of doctor who is not provided with a complete education certificate, *(schulzeugniss der Reife—Testimonium maturitatis.)* The consequence of this regulation is, that the numerous body of physicians of the higher class must have a learned preliminary education; inasmuch as the obtaining of this certificate requires not only a thoroughly-grounded knowledge of Latin and Greek, but also of mathematics, physics, history, and the modern languages; all of which cannot well be acquired, even by the youths of the best parts, before the completion of their nineteenth or twentieth year. French is taught in all the schools, and English and Italian in most.

It is sufficiently evident from all this how well the state has consulted the real dignity of the medical profession, and that this principle has not been lost sight of in the formation of the medical constitution of Prussia, —viz. that none but really liberally instructed physicians can promote the progress of medical science, or take a lead in the noble art which they profess. And certainly the state has a perfect right to demand the preliminary qualifications above mentioned from the candidates for the doctorate; inasmuch as there is no other nation in which so much care is taken in promoting elementary education, and none in which access to the seminaries of instruction is made so easy even to the poorer classes, as in Prussia, where the annual school-fees in the colleges are only twenty dollars, (3*l.*) Of these colleges, all furnished with ample means for instruction, there are in the whole kingdom 124, in which the number of teachers is 1,334; and these colleges, in the year 1832, were attended by 24,461 scholars. The number of these last that left the colleges for the university, in the summer session of 1832, with the *testimonium maturitatis,* was 738; and it may be calculated that, in all, about 1,400 scholars leave the colleges every year with these testimonials; of which number, about one seventh or one eighth part devote themselves to the study of medicine. Cases occur where medical students, intending to undergo the examinations for the doctorate, do not bring with them from college this necessary document; but still the law holds good here as in other cases. Such persons are compelled to give in the *testimonium maturitatis* during the period of their university studies, although it may be inferred that the knowledge acquired at this latter period is not so complete as in the early periods of education.

The legal period of study at the university for the candidates for medical honours, is four years: in future, this will probably be extended to five. For the acquisition of the degree of doctor, the following academical examinations are indispensable: 1. The *philosophical examination* (examen philosophicum) in chemistry, physics, zoology, botany, mineralogy, and philosophy, by a board composed of members of the Faculty of Philosophy. This examination, the object of which is to prevent the students from neglecting the study of the natural sciences, as would otherwise be likely enough to happen, may take place after the two first years of study. 2. The *tentamen medicum,* which is an examination conducted both in writing and *viva voce,* before the dean of the Medical Faculty. By this examination, the dean ascertains the capacity of the candidates to undergo the principal examination. 3. The *examen rigorosum.* This, like the preceding, is conducted in Latin, and takes place before the Medical Faculty, in all branches of medicine. 4. The composition of an inaugural dissertation in Latin, on any subject chosen by the candidate. 5. The public defence of this dissertation.

It is proper here to observe, that the possession of the degree of doctor does not, in Prussia, necessarily comprehend the right to practise; which right can only be obtained after submitting to certain official or state examinations, with which the universities have nothing to do. But, previously to noticing these, it is necessary to say a few words respecting some other establishments for medical instruction.

### I. THE MEDICO-CHIRURGICAL FREDERICK-WILLIAM'S INSTITUTION.

This, formerly the Medico-Chirurgical Pépinière, was established in the year 1795, and is the elementary school for the higher class of army physicians, who are all edûcated here, or, at least, with rare exceptions, and these occurring only during war. In this institution there are at present ninety-one students, who are found with board and lodging at the public cost, and occupy a large building set apart for this purpose. They study here for four years, and are under a bond to serve afterwards in the army for eight years. Into this institution only those young men are received who, besides possessing the general requisites for military service, bring with them the *testimonium maturitatis.* These military students follow the complete course of university studies, with more constant reference to surgery, the knowledge of which is so essential in their case: they attend the same lectures as the other students of the university; the only difference in their case being, that the lectures are regularly repeated to them by the pensionary and staff physicians who reside with them in the institution. No repetitions of this sort, or any other examinations besides those already mentioned, take place in the Prussian universities. In all of these the studies are free, and without any special or restrictive supervision; in which respect they differ considerably from the Catholic universities of Austria.

When a student has completed his four years at the institute, he is placed for a year as surgical house-pupil *(Chirurg,)* in the Charité Hospital; a very great advantage in itself, and still further valuable to him, as it reckons as one of the eight years of service in the army. Most of these military students take the degree of doctor before they enter the army, and, while they serve there, the most deserving are promoted to the higher offices. These higher offices are those of regimental-physician and physician-general; but, before any one can attain to these, he must have served a certain time (not fixed), first in the Guards (*Garde*), as surgeon of a company or squadron, and then, being advanced according to seniority of service, in the Institute and Charité, (part of the time in each), as pensionary-physician and staff-physician, *(Pensionnair und Stabsärtze.)* During this latter period they are occupied in carrying on their studies, for which they have excellent opportunities, as well in the *repetitions* with the students of the Institute, as in the performance of their duties as resident surgeons in the hospital. During the same period they undergo their probationary or state-examinations, which are as indispensable in their case as in that of the civil physicians.

Of medical officers thus attaining the higher stations, there are in all thirty-four, —viz. ten staff-physicians, twelve pensionary-physicians, and twelve surgeons of the Guards; and of these, the senior staff-physician always obtains the first vacant office of regimental-physician. The older unpromoted members of the Institute commonly accept their discharge from the service; if they do not choose to become physicians of battalion, having previously undergone the necessary state-examinations.

The Institute is richly endowed with every thing necessary to its ends, and possesses all the collections and instrumental apparatus necessary for instruction, and particularly a very rich medical library. It is under the authority of the first staff-physician-general of the army for the time being, and a director resident in the institution: the former, at present, is Von Wiebel, body physician to the king; the latter, Physician-general Schulz.

### II. THE MEDICO-CHIRURGICAL SCHOOLS FOR THE EDUCATION OF SURGEONS.*

Beside the universities which are calculated for the education of the more cultivated and learned physicians, the true heads of the profession, and the Frederick-William Institution, which, in a scientific point of view, may be regarded as connected with the University of Berlin, there exist in Prussia other establishments at which persons of a somewhat lower rank in society, the surgeons of the first and second class, as they are named, may receive instruction. From these, the *testimonium maturitatis* is not required, and consequently they can attain neither the doctorate nor any of the higher official stations; and they are restricted by the laws and regulations to a more limited range of practice than the physicians. At present in Prussia, and long since in Bavaria, where a like class of professional men, under the name of *country-doctors* (landärzte) exists, there is a pretty general conviction that surgeons of this kind are necessary and beneficial to the inhabitants of small towns, and of the country generally; and, on this account, their education has been encouraged. Nevertheless, opinion in the country still varies on this point; many being apprehensive lest the increasing number of the less-instructed class of medical men should too much confine the operations of the more instructed in the class of physicians.

### III. THE MEDICO-CHIRURGICAL ACADEMY FOR THE ARMY.

This institution was founded in Berlin in 1811. It has several salaried professors, who however belong also, with few exceptions, to the university. Its object is the education of under-surgeons (Unterchirurgen,) who may attend the lectures given in it, during a period of three years, free of expense, and who, in consequence of this privilege, are bound to serve for three years in the army as Company or Squadron Surgeon. They are very fully instructed in Anatomy, Physiology, Surgery, and everything relating to practical medicine. Their superiors, the Pensionary Physicians of the Frederic-William's Institution, repeat to them the lectures which are delivered in the university. In this establishment there are at this time forty-eight students. Since 1811, there have been in all 499, of whom 308 have entered the army. When, in course of time, they have passed their probationary examinations, they may rise to the rank of Battalion Physicians, if they remain in the army. The greater number of young men, who are well educated, yet unable from deficiency of means to obtain the *testimonium maturitatis*, select this institution as their school; and, when eventually they attain a more fortunate position, they take the necessary steps to obtain the doctorate. The Academy is under the authority of the War Office and immediately under the direction of Ch. W. Hufeland and Von Wiebel. The present professors are:

| | | |
|---|---|---|
| 1. Von Graefe | 6. Link | 11. Turte (Physics) |
| 2. Horn | 7. Mitscherlich | 12. Wolf (Philosophy) |
| 3. Fr. Hufeland | 8. Osann | 13. Eck |
| 4. Kluge | 9. Rust | 14. Reich. |
| 5. Von Könen | 10. Muller | |

### IV. THE SURGICAL PHARMACEUTICAL STUDIUM IN BERLIN.

This is likewise an institution in Berlin, (Rust is the director,) in which young men, who wish to prosecute the study of surgery and pharmacy, are provided with the necessary preparatory information, and are thence admitted to hear the lectures in the University. Particular teachers are not appointed to it, which would be superfluous, the great number of teachers in Berlin being considered. This institution differs from the provincial surgical schools, about to be mentioned in this respect, that no *repetitions* take place in it. In the year 1834, 142 pupils entered, and 123 left this Institution.

* Medicinisch-Chirurgische Lehranstalten oder Chirurgen-Schulen.

### V. PROVINCIAL MEDICO-CHIRURGICAL SCHOOLS.

Of still more recent establishment are the Medico-Chirurgical Schools in the provinces, viz. in Münster, Breslau, Griefswald, and Magdeburg. In these, Anatomy, Surgery, Medicine, Midwifery, are taught in such wise as to fit the students, after three years, to undergo their examinations as surgeons of the first class. They are amply provided with anatomical theatres, clinics, collections, &c., and can even boast of teachers of Latin and the sciences. They differ essentially from the universities in this, that the lectures in them (the schools) are repeated to the students, by repeaters especially appointed for the purpose.

*School of Munster.* This is the oldest of the provincial schools, having been founded in 1821. It makes use of the City hospital, into which, in a period of five years, about 3000 patients are admitted. It is pretty well attended, and has the following teachers, viz. Roling, Busch, Haindorf, Pellengahr (director of the clinical department), Klovekorn, Tourtual, Klatten, Wirtensohn, Riefenstahl (prosector), Waldeck and Becks; the two last for languages and the sciences, unconnected with medicine. It is to be observed that there is also in Münster an University with two faculties, one Roman Catholic and one Philosophical, with which the medical Institution has no connexion.

*School of Breslau.* This was opened in the year 1823: it is connected with the university of that place, and makes use of its establishments. The director is Wendt; and the teachers are Otto, Wentzke, Betschler, and Goeppert; Kannegiesser and Schummel superintend the non-medical part of the institution. The repeaters are Barkow, Remer, jun., and Burchardt. This school is also much frequented, and supplies the wants of the very populous province of Silesia; and from it will gradually be furnished even the poorest districts, which formerly had little or no medical assistance.

*School of Magdeburg.* This was founded in 1827, and has since then educated 273 pupils. At the present time it has 78. In the anatomical theatre there are annually from forty to fifty bodies used in dissection, or for operations; the City Hospital, with 250 beds, offers excellent opportunities for clinical instruction; while the Polyclinic flourishes amid the crowded population of the city. The Obstetrical clinic is only available during the summer session of six months, during which time from fifty to fifty-five labours are obtained for purposes of instruction. The teachers are the following:

1. Andreae, Pathology, Semeiotics, Therapeutics, and Ophthalmology.
2. Brüggemann, Anatomy, Physiology, and Pathological Anatomy.
3. Dohlhoff, . . Surgery and Clinical Surgery.
4. Fritze, . . Materia Medica.
5. Michaelis, . . Natural Sciences.
6. Niemeyer, . . Medical Jurisprudence, Clinical Medicine.
7. Scheibler, . . Surgery.
8. Voigtel, . . Midwifery.
9. Fentzsch, . . Languages.

*Repeaters:* Faber, Schulz, and Varger.
*Directors:* Andreae, Fritze, and Scheibler.

*School of Griefswald.* This is of more recent establishment still: it is united to the university of the same place, and is supplied with professors from it. These are

1. Mandt (Director), Surgery.
2. Schultze, . . Anatomy and Physiology.
3. Berndt, . . Medicine, Clinical Medicine, Midwifery.
4. Seifert, . . Pathology and Therapeutics.
5. Hornschuch, . Natural Sciences.
6. Schoemann, . Languages.
7. Hunefeld, . . Chemistry.

*Repeaters:* Laurer and Biel.

### OF THE CLASSIFICATION, AND FINAL OR STATE EXAMINATIONS OF THE MEMBERS OF THE MEDICAL PROFESSION IN PRUSSIA.

### 1. *Graduated Physicians.**

There are two classes of these: 1. such as profess and practise both medicine and surgery; 2. such as practise medicine only.

First Class (for both medical and surgical practice.) They must have obtained the degree of M. D., according to the legal forms, in some university, domestic or foreign, and subsequently have undergone the final or state examinations (*staatsprüfungen.*) These constitute a sort of probationary course, consisting of the following parts:

1. *The Anatomical Examination.* In this the candidate has to undergo four different trials: *a*, to demonstrate, on the subject, one of the great cavities, (head, chest, or abdomen,) as regards the form, position, relation, and connexion of the contents; *b*, to describe an anatomical preparation, made by himself under inspection; *c*, *d*, to explain two other preparations in sphlanchnology, neurology, angiology, or osteology, at sight.

2. *The Chirurgical Examination.* The object of this is to test the candidate's knowledge of the manual or operative part of surgery, and to this end he has to undergo the following trials: *a*, he must give in a written surgical exercise on a prescribed subject, must state publicly the substance of it, and perform the operation itself on the dead body; *b*, he must discuss (*disseriren*), *ex tempore*, a question in operative surgery,—must state the principal methods of operating,—must demonstrate his knowledge of the instruments,—and finally perform the operation on the dead body; *c*, he must deliver an *ex tempore* dissertation on a given subject, relating to fractures and dislocations; must point out the proper mode of treatment on the model, and apply the bandages.

3. *The Clinico-Medical Examination.* This lasts fourteen days, during which period the candidate treats two patients, under the inspection of the examination-commission; records the history of the disease in the Latin language; and, in the event of a fatal termination, adds the appearances on dissection. During the whole of this time he is daily examined in pathology and therapeutics.

4. *The Clinico-Surgical Examination.* This likewise lasts fourteen days, and the candidate takes the charge of two surgical patients. The history of the cases is recorded in German, and the daily examinations are on the various phenomena presented by the cases.

5. *The Final or viva-voce Examination.* This takes place in public, and extends to every department of medicine. It is conducted in the German language, by four of the commissioners, and, in general, lasts about three hours.

Second Class (for medical practice only). The candidates in this class have to undergo the same examinations as the former, with the exception of that for operative surgery. Surgical knowledge, however, is equally expected in them as in the others: consequently, they undergo the clinical trials, and, in the oral examinations, are equally tested in surgery; the only difference in both cases being the omission of the operative part.

The Graduated Physicians are the only members of the profession in Prussia who are qualified to fill any medical office, from the lowest, that of *Kreisphysicus* (*quasi* district-doctor), up to the highest, that of *Geheimer Ober-Medicinalrath* (High-Medical-Privy-counsellor.) To qualify, however, for these offices, two other examinations are still requisite, viz. (1) the *obstetrical*, which may take place before any of the medical colleges in Berlin or in the provinces; (2) the *forensic or jurisprudential* (physicats-prüfung), which consists in the composition of four exercises or treatises on given subjects in some of the branches of state medicine, and in an oral examination in the

* *Promoverte practische aerzte.* The word *Practische* distinguishes the physicians who practise from those who devote themselves to teaching in the universities.

same. This last is undergone only two years subsequently to the probationary trials, and in case the candidate has not exhibited proofs of some particular excellence at that time. To the higher posts in the army, from regimental physician upwards, only physicians of the first class can be appointed,—i. e. such as have gone through all the surgical examinations with credit.

2. *Surgeons of the First Class.*

These must possess the necessary knowledge of Latin, and have studied three years in some medico-chirurgical school. They must undergo precisely the same examinations as the graduated physicians, with the exception that their exercises in the clinical department are not in Latin; and that, generally, as well in these as in the final oral examination, they are more tested as to practical knowledge than as to literary acquirements. They are not qualified for the higher medical appointments in civil practice; they can only become *Kreischirurgen*, (district-surgeons;) or, at most, *assessors in the medical colleges*, (assessoren an medicinal collegien); and in the army they cannot rise higher than battalion-physician. They are entitled to practise medicine, but not in towns where there is already any graduated physician: in such places they are restricted to the exercise of surgery; a circumstance which induces many, to the great advantage of the public at large, to settle in situations where there is no physician.

3. *Surgeons of the Second Class.*

These are authorised to practise what has been termed *lesser surgery*, also to treat wounds, fractures, and luxations; and they pass examinations to this end at the provincial medical colleges. They may be regarded in the light of assistant surgeons, and constitute a subordinate, but on the whole a necessary and useful body of practitioners. They are restricted from treating internal diseases and from midwifery.

THE SUPERIOR BOARD OF EXAMINATION.

A particular Board or Court with this title (Die Ober-Examinations-Commission,) is established in Berlin, for the probationary examination of candidates. It is at this time directed by Klug, but is under the immediate authority of the government. The present members are:—For the anatomical examination, Müller and Eck; for that in operative surgery, Wagner and Kothe; clinical surgery, Trüstedt and Jüngken; clinical medicine, Bartels and Wolf; and for the final *viva-voce* examination, Link, Osann, Hecker, Busse, Grossheim, Albers, Dieffenbach, and Mitcherlich. According to the regulations, all candidates for the rank of physicians or surgeons of the first class must undergo their examinations in Berlin; but, as it would be severe and unjust to oblige the poorer individuals to submit to the expense of residing in Berlin for half a year, (and the probationary course of examinations cannot well occupy less than this period,) an exception is made in their favour, by which they may undergo their examinations in the medical colleges of Breslau, Magdeburg, or Coblenz. These examinations take place upon the same scale as at Berlin; and, in order to obviate any want of uniformity that might arise, they are all under the control of government.

OF THE NUMBER AND DISTRIBUTION OF THE MEDICAL PROFESSION IN PRUSSIA.

In the year 1833 there were, in Prussia, 2159 graduated physicians, 466 surgeons of the first class, and 1846 surgeons of the second class, making, in all, 4,471; in which number were included 1784 accoucheurs. Besides these, there were 515 apothecaries of the first class, 727 apothecaries of the second class, 17 veterinary surgeons of the first class, and 180 veterinary surgeons of the second class. There were, moreover, 10,766 midwives, all properly instructed and licensed; and none other are allowed to practise.

The distribution of the members of the medical profession is very unequal, depending mainly on the affluence and industrial activity of the several provinces. In the whole kingdom, in 1833, then possessing a population of 13,099,803, there was one physician or surgeon for every 2,929 inhabitants. In Berlin there is a disproportionately large number of medical men, no less than one for every 776 inhabitants; whilst, on the other hand, in the province of East Prussia, one has to take care of the health of 6,025 individuals. So great a disproportion as this certainly exists in no other province, and, indeed, Berlin might spare some part of her medical treasures to the others, without any material loss. In West Prussia, the proportion is one medical man in 5,539 inhabitants; in Pomerania, one in 3,564; in Brandenburg (exclusive of Berlin), one in 2,915; in Posen, one in 4,838; in Silesia, one in 2,924; in [Prussian] Saxony, one in 1,916; in Westphalia, one in 2,677; in Cleves and Berg, one in 2,361; in Niederrhein, one in 3,796.

In general,—still keeping out of view the superfluity of Berlin,—we may observe a satisfactory increase of the members of the medical profession, keeping pace with the increasing population; and although it is true here as every where else that the greater number of professional men settle in the large towns, still there is an obvious improvement in the relative proportion of medical aid to the inhabitants, not only in the smaller towns, but in country places, so that even in this particular, the fitness for its purpose (zwechmässigkeit), of the Prussian medical constitution, is conspicuous over that of the neighbouring states.

### MILITARY MEDICAL OFFICERS.

In the foregoing statements, the superior military physicians are included, as they all enter into private practice. The arrangements in the army itself are as follow:—Von Wiebel, first *Staff-physician-general* of the army, is at the head of the whole military medical establishment (there are only three others of the same rank, *viz.* Büttner, Von Gräfe, and Rust). Each of the nine divisions of the army has one *physician-general* of division, and each regiment of the standing army, and every brigade of artillery, has a *regimental physician.* Of this last class, there are at present ninety-two; *viz.* forty-four for the infantry regiments, one for the jager-guard battalion, thirty-eight for the cavalry regiments, and nine for the artillery brigades. Thirty-six infantry regiments have three battalions, the regimental physician taking charge of two, and a battalion-physician the third. Two battalion-physicians of the guard have the title of regimental-physician. Eight infantry regiments, with only two battalions, have no battalion-physicians. The militia (Landwehr, an extensive corps,) has no regimental physicians, but physicians of battalion merely, and only one for each battalion-squadron; and of these there are, in this species of troops, somewhere about 152. There are besides, in the large towns and fortresses, garrison staff-physicians appointed, possessing the same rank as battalion-physicians. All military physicians of this rank must have undergone the medical probationary examinations. The inferior medical departments of the army are filled by company or squadron-surgeons, each company and squadron of the standing army having one. Every infantry regiment of three battalions, and every artillery brigade, has twelve of these surgeons, and every cavalry regiment four. The probationary state examinations are not required of these, and they are restricted from engaging in private practice. For the accommodation of the sick, each regiment, during peace, has its own hospital; during war, other arrangements take place.

### CIVIL MEDICAL FUNCTIONARIES.

A great number of medical functionaries (Aertzliche Beamte.—Medinal-beamte) are kept in the pay of government, for the purpose of giving opinions in cases having reference to medical police, sanatory regulations, and state medicine, and of assisting the courts in general relating to their department. Every circle or district (kreis), of which there are, in all, 335, has a *kreis-physicus* and a *kreischirurgus:* they both live in the chief town of the district, and besides their salary, which, for the first, is on the average 200 dollars, have their travelling expences defrayed.

Each of the twenty-five governments into which the kingdom is divided, has a *government medical counsellor*, (regierungs-medicinalralh,) for the cognizance of medical cases coming under the notice of the administration, each with a salary of from 900 to 1200 dollars (£135 to £180). To this functionary the *Physici* of the district give in their reports, and from him emanate all the sanatory police arrangements.

Lastly, each of the provinces of the kingdom, eight in number, is provided with a *medical college*, composed of one government medical counsellor, a certain number of medical counsellors, and one surgical and pharmaceutical assessor. These colleges are in Königsberg, Posen, Berlin, Stettin, Breslau, Magdeburg, Munster, and Coblenz, and, besides taking cognizance of the usual cases requiring state interference, are charged with the examination of surgeons of the second class, and also of dentists and apothecaries of the second class. Respecting the examination of midwives, for the instruction of whom in sufficient numbers there are especial schools in several towns, particular regulations exist; and these are so completely effective, that the whole nation is now almost entirely supplied with good midwives. The supreme medical court in Prussia is the *Board for spiritual, educational, and medical affairs* (v. Altenstein), having eight counsellors in the *medical department*, and a *scientific deputation*, consisting of ten members, for reporting on cases laid before it.

### HOSPITALS.

Hospitals exist in Prussia, in a number proportioned to the wants of the public. The greater number are civil establishments, only a few being under the immediate direction of government. Those only are employed for purposes of instruction, which are situated in places in which medical schools exist. In Berlin, the *Charité-Krankenhaus*, founded in the year 1726, and having about 1000 beds, possesses the most extensive clinical establishment. As, according to the municipal government of Prussia, every town regulates its own affairs, the principles on which the different hospitals are governed are naturally very various; and hitherto there is no Central Board, which might place all the hospitals in the kingdom on the same general system. The *Curatorium of hospital affairs*, some years since established in Berlin, has as yet only the *Charité* under its direction, and in regard to all the other establishments of the kingdom, can merely advise or recommend. For this reason it becomes necessary to give an account of each of the principal hospitals separately; and this I purpose doing in my next report. I hope also in my next communication to furnish some details respecting the Medical Societies in Prussia, of which *the Hufelandian Medico-chirurgical Society* (Die Hufelandsche Medicinisch-chirurgische Gesellschaft), and *The Prussian Medical Union* (Der Verein für Heilkunde in Preussen), both in Berlin, and *The Rhenish Society of Natural History and Medicine* (Die Rheinische Gesellschaft für Natür-und Heilkunde) in Bonn, are the most considerable. All these can boast of the fellowship of many English physicians. JUSTUS FRIEDRICH CARL HECKER, M.D.

*Berlin; 23d Nov.* 1835.

---

